# Finger Forces in Clarinet Playing

**DOI:** 10.3389/fpsyg.2016.01140

**Published:** 2016-08-04

**Authors:** Alex Hofmann, Werner Goebl

**Affiliations:** ^1^The Austrian Research Institute for Artificial Intelligence (OFAI)Vienna, Austria; ^2^Institute of Music Acoustics (IWK), University of Music and Performing Arts ViennaVienna, Austria

**Keywords:** clarinet performance, finger forces, articulation, motor actions, timing, sensors

## Abstract

Clarinettists close and open multiple tone holes to alter the pitch of the tones. Their fingering technique must be fast, precise, and coordinated with the tongue articulation. In this empirical study, finger force profiles and tongue techniques of clarinet students (*N* = 17) and professional clarinettists (*N* = 6) were investigated under controlled performance conditions. First, in an expressive-performance task, eight selected excerpts from the first Weber Concerto were performed. These excerpts were chosen to fit in a 2 × 2 × 2 design (register: low–high; tempo: slow–fast, dynamics: soft–loud). There was an additional condition controlled by the experimenter, which determined the expression levels (low–high) of the performers. Second, a technical-exercise task, an isochronous 23-tone melody was designed that required different effectors to produce the sequence (finger-only, tongue-only, combined tongue-finger actions). The melody was performed in three tempo conditions (slow, medium, fast) in a synchronization-continuation paradigm. Participants played on a sensor-equipped Viennese clarinet, which tracked finger forces and reed oscillations simultaneously. From the data, average finger force (*F*_*mean*_) and peak force (*F*_*max*_) were calculated. The overall finger forces were low (*F*_*mean*_ = 1.17 N, *F*_*max*_ = 3.05 N) compared to those on other musical instruments (e.g., guitar). Participants applied the largest finger forces during the high expression level performance conditions (*F*_*mean*_ = 1.21 N). For the technical exercise task, timing and articulation information were extracted from the reed signal. Here, the timing precision of the fingers deteriorated the timing precision of the tongue for combined tongue-finger actions, especially for faster tempi. Although individual finger force profiles were overlapping, the group of professional players applied less finger force overall (*F*_*mean*_ = 0.54 N). Such sensor instruments provide useful insights into player-instrument interactions and can also be used in the future to give feedback to students in various learning and practising situations.

## 1. Introduction

Clarinettists control multiple parameters simultaneously to produce a well-sounding sequence of tones. These parameters include the blowing pressure, the lip force, and the lip position in order to produce the sound (Almeida et al., 2013). However, the parameters become more complex in expressive music performances. When changing the pitch of the tones, wind instrumentalists have to make quick and precise finger movements to produce clean note transitions (Almeida et al., 2009). At the same time, the finger movements at the tone holes have to be coordinated with the articulatory tongue actions. For tongued articulation, single-reed players use their tip of the tongue to strike the reed while the blowing pressure is held constant (Liebman, 1989). This allows them to shape the transients of the sound. In a previous study with saxophonists, Hofmann and Goebl (2014) observed that the timing precision of the finger movements had a greater influence on the performance than the tongue, even though the tongue strokes were giving the sound onsets in portato playing. In this study, we aim to verify this finding on another wind instrument, the clarinet.

Measurements with sensors and motion capture technology on the piano showed that the way the finger approaches the piano key had an influence on the produced sound (Goebl et al., 2014) and the timing of the performed tone sequences (Goebl and Palmer, 2008). A percussive touch, produced by a key *struck*, contained different sound components than a non-percussive *pressed* touch, even though for both tones the hammer was hitting the string with the same velocity. Moreover, it was shown that a key stroke with a larger finger-key surface impact improved the timing of the following tone. A follow-up study in the same laboratory looked into finger motion of clarinet students and also observed improved temporal accuracy for clarinettists who used faster finger movements (Palmer et al., 2009b).

On the violin, sound production comes from bowing actions with the right arm that must be coordinated with the left-hand fingerings (Baader et al., 2005). The left-hand finger forces for holding down a violin string were measured to be on average 2.7 N (Kinoshita and Obata, 2009). Kinoshita found that for fast tempi, participants reduced their finger force to 1 N. Passages requiring loud dynamics showed increased finger force up to 5 N on average. A study on the guitar reported that guitarists applied nearly ten times more finger force (30–50 N) to hold the guitar strings properly to the fingerboard, hereby the applied force directly influences the sound quality (Hori et al., 2013). On the clarinet the fingers control pitch by closing and opening tone holes. Clarinet teachers recommend to spend only minimal finger forces to close the tone holes airtight (Wehle, 2007). These minimal forces depend on the particular characteristics of the levers and springs in the key work. We expect therefore that professional clarinettists are applying less force to the tone holes than string instrumentalists to the vibrating strings to keep them tight to the fingerboard.

Observations of the body movements of clarinettists during performance showed that also their body movements were related to the musical context (Palmer et al., 2009a; Caramiaux et al., 2012). The circular bell movements, the flapping with the arms and the bending at the waist were similar for repetitions of the same piece and in relation to the musical expression of the performance (Wanderley et al., 2005). Although the sound production on the clarinet is happening at the mouthpiece and the function of finger-actions on the clarinet is merely to open and close the tone holes, such expressive gestures may also influence the finger forces applied to the instrument. We hypothesize that musical situations (dynamics, tempo, and register) have an influence on the finger forces applied to the tone holes. Furthermore, the hands are also holding the instrument. This requires balancing the instrument on the thumbs without gripping it with the other fingers. Imbalanced stress to the thumbs has already been identified as a reason for overuse syndromes with clarinettists (Nemoto and Arino, 2007; Diethelm, 2011). Overuse syndrome is a general problem for professional music performers across various instruments (Altenmüller and Jabusch, 2004). It is of interest for musicians, music teachers and medical personnel to gain insight into force related player-instrument interactions. By measuring the finger forces at the keys of the clarinet we aim to better understand how clarinettists' fingers interact with the instrument.

## 2. Materials and methods

In an empirical study, clarinet students and professional clarinettists played under controlled performance conditions. Their articulatory tongue actions to the vibrating reed and the applied finger forces to the tone holes were measured throughout the experiment. The experiment consisted of two tasks. The first task is a performance task, with a focus on examining the finger forces under different musical playing situations. The second task is a repetition of the technical exercise task from a previous study on the saxophone (Hofmann and Goebl, 2014) with a modified measurement setup to capture the finger forces at the clarinet.

### 2.1. Stimuli

For this experiment, different musical material was collected to test our hypothesizes. Eight excerpts from the Clarinet Concerto No.1 by Carl Maria von Weber were selected to fit a 2 × 2 × 2 design (register: low–high, dynamics: soft–loud, tempo: slow–fast). An overview table and the individual score excerpts are provided as Supplementary Table 1 and Supplementary Figures 2, 3. In an additional testing condition, the experimenter instructs the clarinettists to play with varying levels of expression (expression: low–high). For the technical exercise task, the isochronous 23-tone melody from Hofmann and Goebl (2014) was modified to examine the timing on a Bb-clarinet (see Supplementary Figure 3). The first part of the melody (tones 1–8) is a tone repetition, where only tongue-actions are required. The following part requires combined tongue-finger actions (tongue articulation) or only left-hand finger-actions (legato articulation).

### 2.2. Participants

In this study, 10 female and 13 male clarinettists (*N* = 23, mean age = 27 years, range = 19–45 years) from Vienna (Austria) participated. Seventeen participants were students from the University of Music and Performing Arts Vienna. The remaining six participants were professional orchestral or ensemble performers. Five of these professionals were also teaching at an academic institution.

### 2.3. Experimental setup

**Figure 1:**
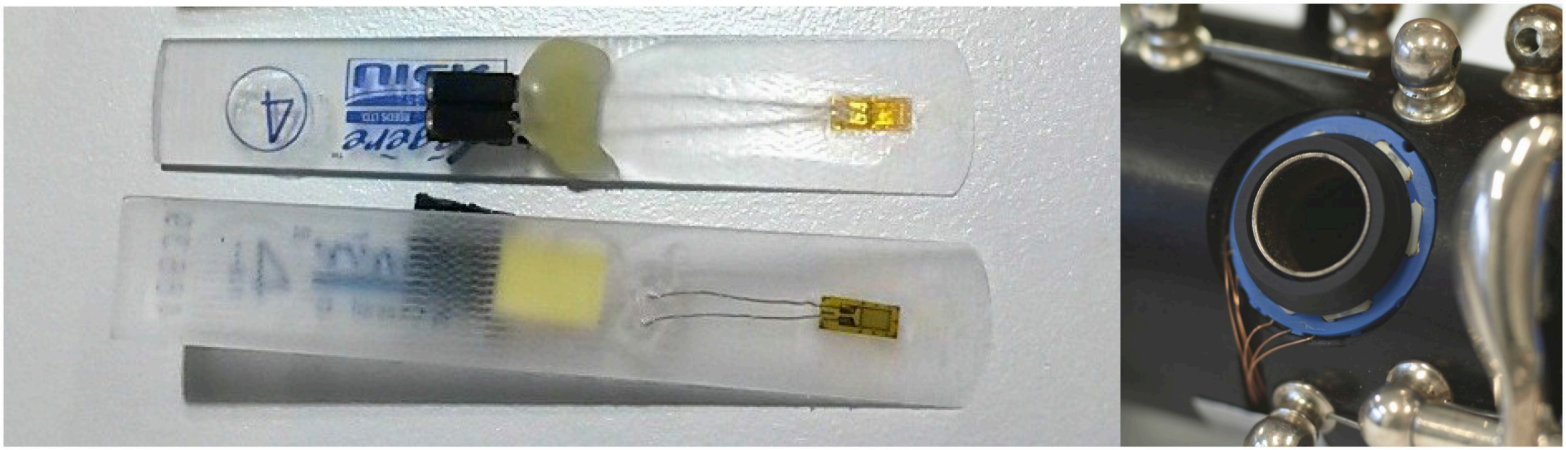
**Left:** Synthetic clarinet reed with a strain gauge sensor to monitor tongue articulation during performance. **Right:** Viennese clarinet with ring-shaped force sensor attached to the tone hole (key-work removed for the picture).

The experimental setup consisted of a Viennese clarinet in Bb (by Martin Foag, Hafenhofen, Germany), with special ring-shaped force sensors (see Figure 1, right) attached to the six main tone holes of the instrument (Weilguni, 2013). Each sensor ring contained three measurement cells which captured the load from the finger applied to the sensor when a tone hole is closed. Synthetic clarinet reeds (by Légère, Canada) were equipped with strain gauge sensors to measure the bending of the reed during performance (see Figure 1, left). This enabled us to monitor the articulatory tongue-reed interactions of the performers (Hofmann et al., 2013). The sensors and a microphone (by DPA, Denmark) were connected via BNC-cables to a multi-channel recording device (by National Instruments, USA). The multi-channel data was simultaneously recorded onto a computer hard disk using a sampling rate of 11,025 Hz (16-bit).

### 2.4. Procedure

The experiment was conducted in accordance with the Declaration of Helsinki: In the beginning of the experiment, all participants gave written consent to participate in the procedure. They played under normal performance conditions and received a nominal fee at the end of the experiment. For this experiment, all participants played on the same sensor clarinet, but were allowed to use their own mouthpiece. Each participant chose a sensor reed with the preferred reed strength (between 3.5 and 4.75) and had 5 min to warm up with the instrument. The experiment consisted of two performance tasks: first, the eight excerpts from the Weber Concerto were performed under two different conditions of expression level (low–high). For the low expression level condition, participants had to perform together with a quarter note metronome click and were instructed to focus on technical aspects of playing (correct tones, precise rhythm, and good intonation). In contrast, for the high expression level condition, the experimenter muted the metronome click and encouraged the participants to play with a high level of musical expression, similar to a concert performance situation. We recorded two trials per expression condition. In total, 2 (expression levels) × 2 (trials) recordings were made for each of the eight excerpts. Subsequently, the participants filled in a questionnaire on their musical background. As the final task, the participants played the technical exercise. In this task, the isochronous 23-tone melody had to be performed with three articulation techniques (legato, portato, and staccato articulation) under three different tempo conditions (slow inter onset intervals = 250 ms, medium IOI = 178.6 ms, fast IOI = 144.2 ms). For each recorded trial, the players synchronized with the metronome for the first two repetitions of the melody. Following a synchronization-continuation paradigm, the metronome was then muted and the clarinettist continued playing until the melody was repeated 6 times in total. We recorded two trials per tempo condition. The entire procedure lasted for about 50–60 min per participant.

### 2.5. Data processing

**Figure 2:**
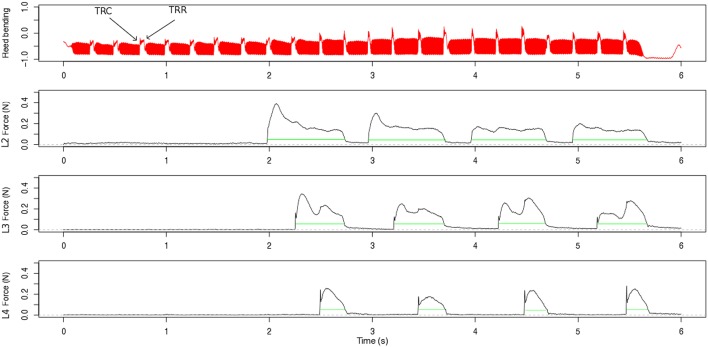
**Excerpt of the data captured in the technical-exercise task with portato articulation in the slow tempo condition for one participant (ID 20)**. The top panel shows the reed-bending signal. Two landmarks, one for a tongue-reed contact (TRC, note offset) and one for a tongue-reed release (TRR, note onset) are labeled. The panels below depict the captured finger forces at the tone holes, for the (L2) left-hand index finger, (L3) middle finger, and (L4) ring finger. The green lines indicate the detection of when the finger pressed the force sensor ring.

For the finger force sensors, calibration data was provided by the developers to convert the captured voltages of the sensors into force in Newtons (details about the properties of the sensors can be found in Weilguni, 2013). The force profiles of the three measurement cells were added to a compound force profile per sensor ring. To demonstrate the captured signals, Figure 2 shows an excerpt of the reed-bending signal together with the measured finger force profiles for the left-hand, for one participant who performed the technical exercise task with portato articulation in the slow tempo condition. Regions were selected automatically from the unfiltered compound finger profiles, starting as soon as the sensor measured a force until its release, which may occur several tones later depending on the score and the fingering (green line). We calculated the mean finger force (*F*_*mean*_) and the maximum finger force (*F*_*max*_) over these regions for each finger and each recorded trial (see Figure 2). As the finger has to overcome the static force by the spring of the key-work around each of the tone holes, the individual static force of each spring was measured (0.1–0.4 N) and added to the finger force profiles to estimate the true finger force exerted by the player.

It turned out during the experiment that the design of the finger force sensors was challenged by the present real-world application. The three measurement cells were glued with conductive silver-epoxy adhesive into each sensor ring (Weilguni, 2013). This rigid fixation was not able to handle the stress over the entire experiment and caused occasional artifacts. These artifacts were clearly visible in the raw signal as regions of peak values. All captured data were carefully inspected manually and affected cell signals removed of all data of that participant. As long as one or two cells were affected, the force profile was extrapolated from the remaining cell(s). Only in the case of sensor 4 (right hand index finger), where the main sensor fixation broke after participant 6, all three cells contained artifacts and no finger force profiles were available. As a consequence, one of the six finger force profiles at the performance task were unavailable for subsequent analysis. For the technical exercise task one participant's data had to be excluded from the analysis.

## 3. Results

### 3.1. Finger forces in clarinet playing

In the performance task, participants performed eight different excerpts from the Weber Clarinet Concerto. These excerpts consisted of a variety of typical situations in practising clarinet playing and performing clarinet music. Overall, the measured average finger forces of all participants were (*F*_*mean*_ = 1.17 N, *SD* = 0.37), and showed no difference between participant group (professionals, students) or sex. Nevertheless, the peak finger forces of the individual players varied between *F*_*max*_ = 0.84 N and *F*_*max*_ = 12.95 N, but were on average *F*_*max*_ = 3.05 N. To examine the finger forces under different levels of musical expression (expression: low–high), the participants were instructed by the experimenter on how to play before the recording of each trial. A one-way repeated measures analysis of variance (ANOVA) on the mean finger force (*F*_*mean*_) by expression level condition, showed a significant effect of the expression level $\left[F_{\left(1,22\right)}=26.2,p<0.001,\eta^{2}=0.74\right]$. The measurements showed that the finger force were higher for the high expression level condition (low expression: *F*_*mean*_ = 1.13 N, high expression: *F*_*mean*_ = 1.21 N).

**Figure 3:**
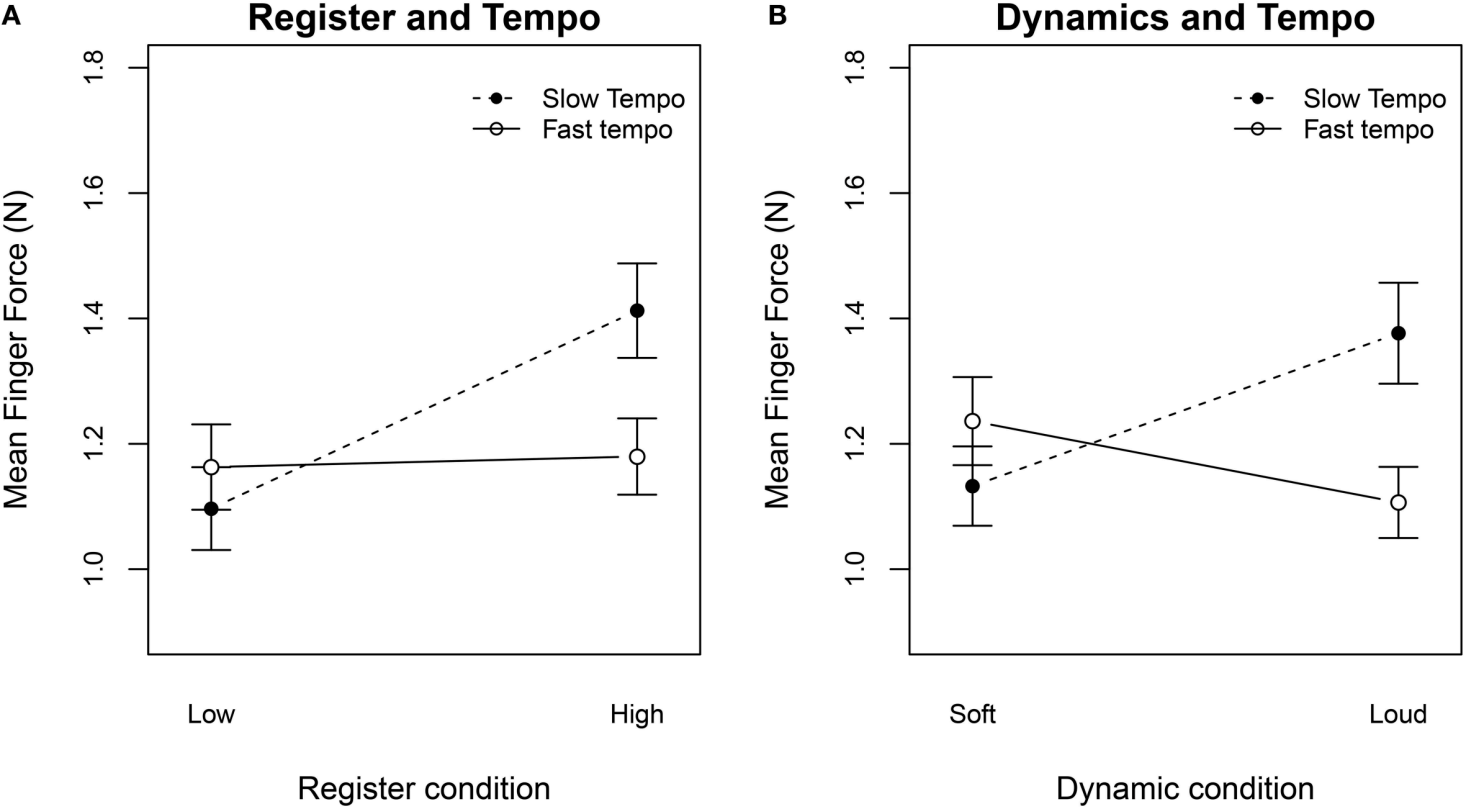
**Finger forces measured (*F*_*mean*_) for the high expression level performances of the Weber Concerto excerpts**. Graph shows the interactions of the register, the dynamics and the tempo. **(A)** For slow tempi, participants' finger forces increased when playing in the high register, whereas for the fast tempi conditions the finger forces remained constant (solid line). **(B)** Louder dynamics resulted in increased finger forces for the slow tempo condition but showed an opposite effect for the fast tempo. The error bars show the standard error of the mean.

From the eight different excerpts we were able to investigate the finger forces applied for different registers (low–high), tempi (slow–fast), and dynamics (soft–loud) when playing expressively (see Figure 3). A three-way repeated measures ANOVA on *F*_*mean*_, by register, tempo, and dynamics, indicated three significant effects (register $\left[F_{\left(1,22\right)}=19.04,p<0.001,\eta^{2}=0.681\right]$; dynamics $\left[F_{\left(1,\;22\right)}=4.56,p<0.05,\eta^{2}=0.414\right]$; tempo $\left[F_{\left(1,\;22\right)}=5.22,p<0.05,\eta^{2}=0.438\right]$), as well as two significant interactions (register and tempo [*F*_(1, 22)_ = 18.39, *p* < 0.001]; dynamics and tempo [*F*_(1, 22)_ = 23.52, *p* < 0.001]). Playing in the high register (*F*_*mean*_ = + 0.17 N) or with loud dynamics (*F*_*mean*_ = +0.06 N) significantly increased the applied finger forces to the tone holes. When the participants played sequences in a fast tempo, they applied less finger forces to the tone holes than for the slow sequences (*F*_*mean*_ = −0.08 N). The trend of this observation is in line with the findings Kinoshita and Obata (2009) reported for the violinists, but on a much lower absolute force level. For the technical exercise task, the average mean finger force dropped to a very low level of *F*_*mean*_ = 0.64 N. This finding can be justified by the meeting of several playing conditions that would already indicate light fingering technique: non-expressive playing in the low register with a comfortable (mezzo forte) dynamic level.

### 3.2. Self-evaluation of finger forces

**Figure 4:**
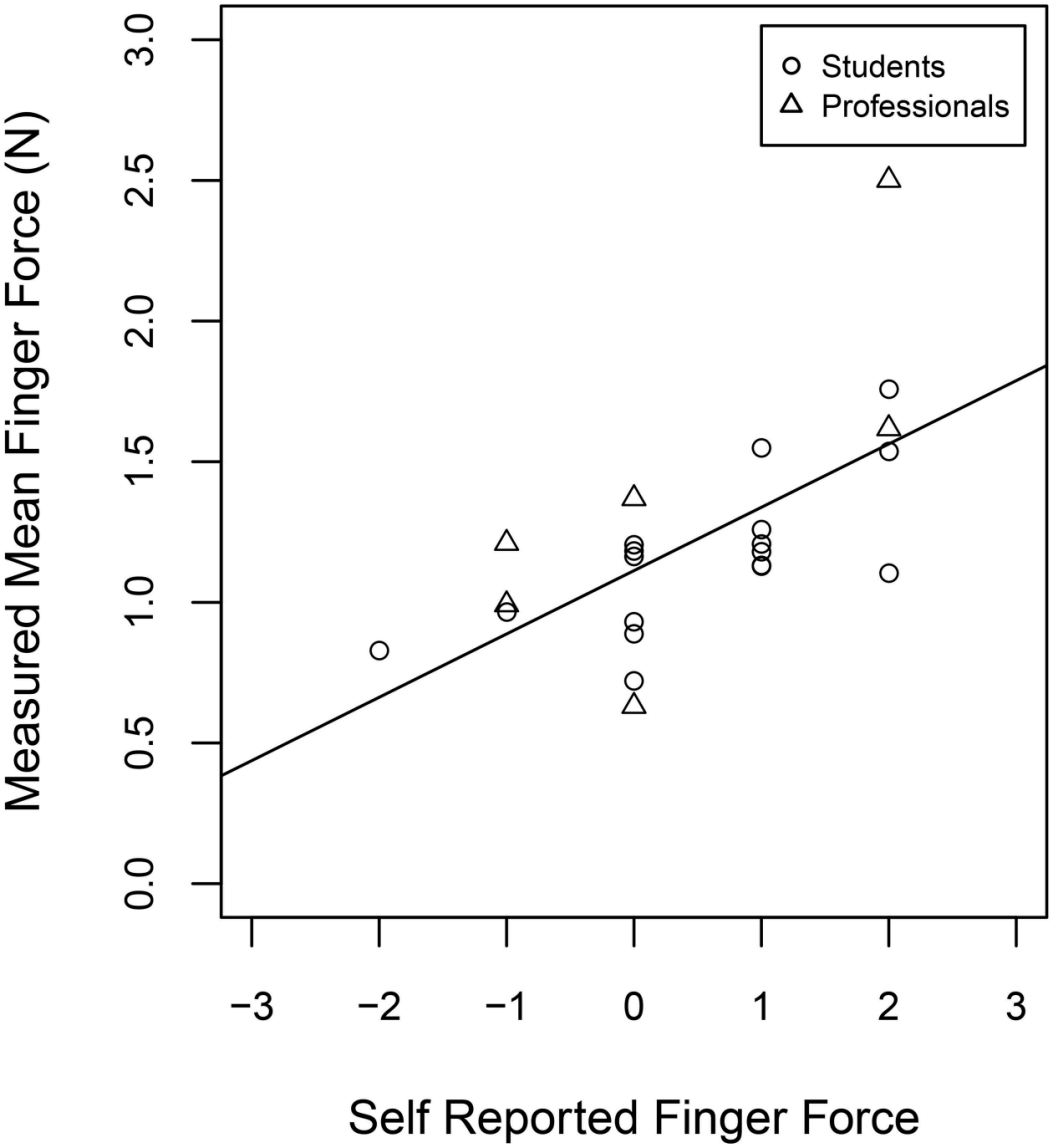
**Self-estimation of finger forces (from −3 to +3) and measured finger forces *F*_*mean*_ for the expressive performances of the Weber Concerto**.

In the questionnaire, participants were asked to self-evaluate their finger forces, without knowing any results of the finger force measurements. Participants had to report on a seven-point rating scale (from −3 to +3). We correlated the measured overall finger force of each participant (*F*_*mean*_, high-expression performance condition trials) with the responses in the questionnaire. The measured and the self-evaluated finger force showed a positive correlation (*r* = 0.64, *p* < 0.001; Figure 4). We were also interested in the physical discomfort of the clarinettists. Therefore, we asked them how often they recognize problems during or after playing. Permanent discomfort in relation to clarinet playing was not reported among our participants, but 19% reported occasional discomfort during playing and 24% reported occasional discomfort after playing the clarinet. Looking into the reported self evaluation of finger force indicated that participants who reported physical discomfort gave a higher self evaluation of finger force (+1.2). Comparing this with the force measurements showed the trend that participants who reported physical discomfort applied larger finger forces during expressive clarinet performance (*F*_*mean*_ = 1.4) than the remaining participants (*F*_*mean*_ = 1.08 N). Although, a one-way repeated measures ANOVA on *F*_*mean*_ with discomfort as between-subjects factor revealed no significant effect of discomfort on the measured average finger forces [*F*_(1, 19)_ = 3.62, *p* = 0.07].

**Figure 5:**
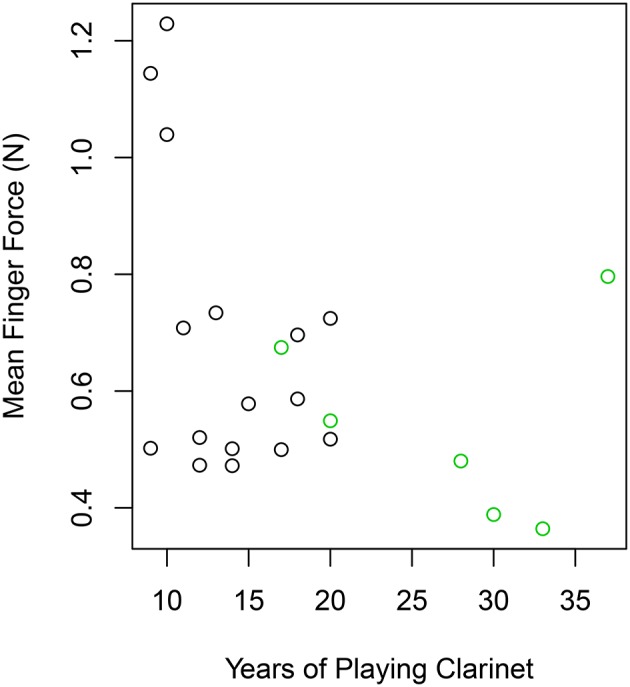
**Mean finger forces (*F*_*mean*_) per participant measured during the technical exercise task in comparison to the experience in clarinet playing in years (green = professionals, black = students)**.

Furthermore, we were interested if experience in clarinet playing might alter how much finger forces participants apply to the instrument. In the questionnaire we asked the participants for how many years they played clarinet and correlated their responses with their average finger forces (*F*_*mean*_) measured during the technical exercise task. The results showed that the correlation did not reach significance (*r* = −0.39, *p* < 0.076) to the standard alpha level of 0.05, but the trend of applying less finger force with longer experience in playing the clarinet is visible in Figure 5.

### 3.3. Timing in the technical exercise task

To analyse the timing of the performances, landmarks in the reed signal were detected when the tongue contacted the reed (TRC, note offset) and when the tongue released the reed (TRR, note onset). The detection algorithm is based on wavelet methods for time series analysis (Percival and Walden, 2006)^1^. From the note onset landmarks, we calculated inter-onset intervals (IOI) by finding the time distance between the consecutive onsets (*IOI*_*x*_ = *t*_*x*+1_ − *t*_*x*_). To examine the deviation from the given tempo, the timing error was calculated by (*IOI*_*obs*_ − *IOI*_*exp*_)∕*IOI*_*exp*_. A sequence performed too fast results in a negative value, while a sequence played too slowly results in a positive value. The regularity of the IOIs was calculated by the coefficient of variation *CV* = *SD*_*IOI*_∕*Mean*_*IOI*_. A regular IOI sequence results in a value close to 0 and higher variability would increase the CV value. All participants were able to perform the melody with the metronome click (timing error = 0.001; CV = 0.089) in all three tempo conditions (slow, medium, and fast). Two three-way repeated measures ANOVAs for the two timing measures by tempo condition and synchronization condition as within-subjects, and participant group as between subjects factor showed significant effects for the tempo condition (*p* < 0.05) and but no effect between the two groups of participants (*p* > 0.21). The timing quality for professionals and students was in general the same. This might be explained by the fact that all students already successfully passed the music university entrance auditions and played the clarinet on average for more than 13 years.

**Figure 6:**
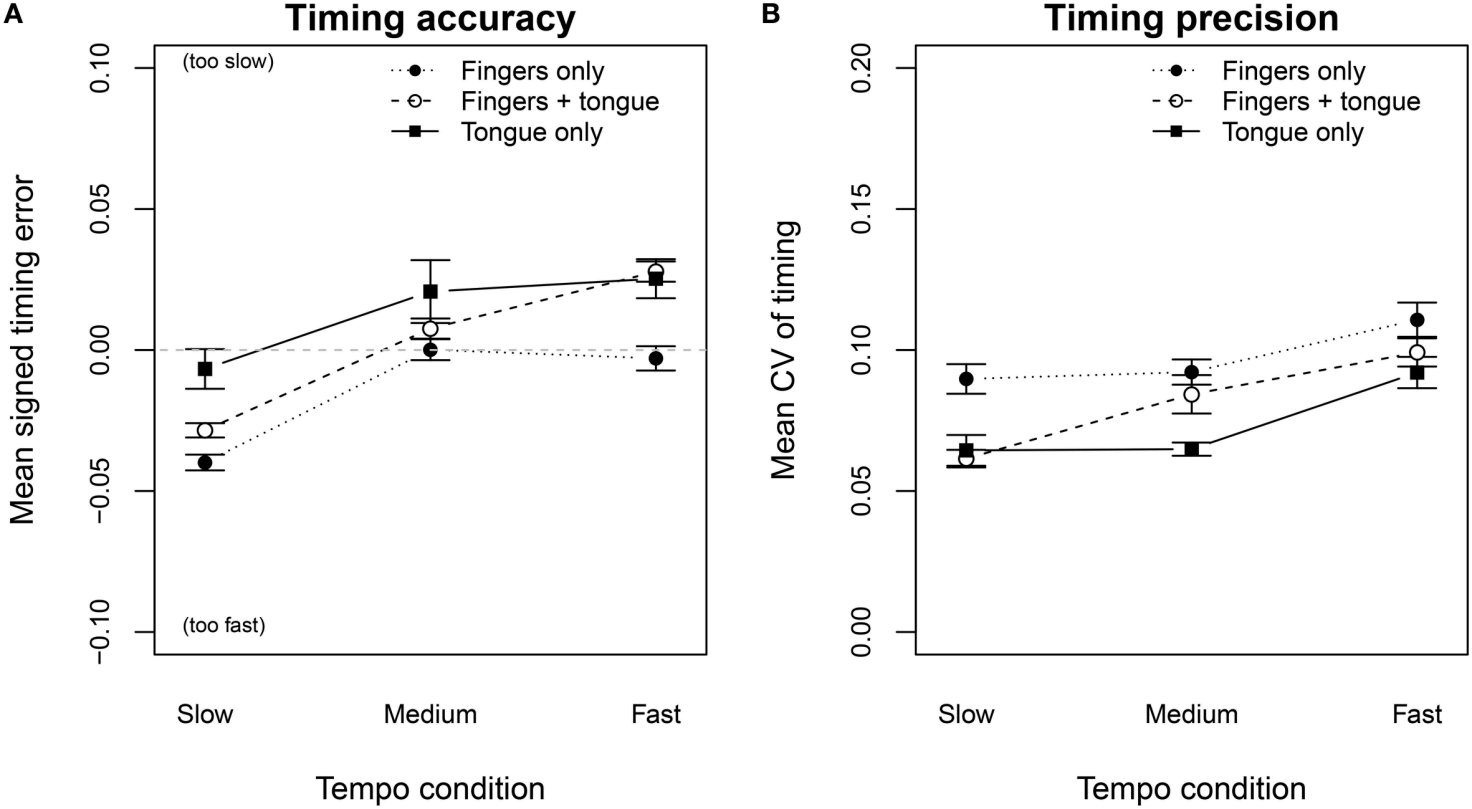
**Timing error (A) and coefficient of variation (B), for the technical-exercise task, played in three tempo conditions, grouped by the effectors required to perform the tone sequence on a Bb-flat clarinet**. Error bars show the standard error of the mean.

In the following section our analysis will focus on the timing of the legato and portato sequences under the continuation condition (metronome turned off). The melody of the technical-exercise task was designed to test three different effector combinations (tongue-only actions, finger-only actions, and tongue+finger actions) to play the note sequence. In one playing condition only left-hand finger actions were required to play the sequence (legato articulation). In the other condition tongue and finger actions had to be coordinated, and in the third condition, the tone repetition, only tongue actions were required. A two-way repeated measures ANOVA on timing error by effector and tempo condition revealed a significant main effect of the effector $\left[F_{\left(2,\;42\right)}=8.95,p<0.001,\eta^{2}=0.547\right]$ and the tempo $\left[F_{\left(2,\;42\right)}\;=\;18.52,p<0.001,\eta^{2}=0.685\right]$, but no interactions. Figure 6A shows the timing error for all three tempo conditions grouped by the effectors. The dotted line depicts the tendency of the participants to play too fast in the slow tempo condition when only finger-actions were required. They played more accurately in the medium and fast tempo conditions. Conversely, with only tongue-actions (black line), the participants tended to slow down in the medium and the fast tempo conditions. Using both effectors in a coordinated fashion (dashed line), the timing error diminished for the slow tempo and the medium tempo, but not in the fast tempo.

The same ANOVA on the timing precision also showed a significant effect of the effector $\left[F_{\left(2,\;42\right)}=6.317,p\;<\;0.01,\eta^{2}\;=\;0.481\right]$ and the tempo $\left[F_{\left(2,\;42\right)}=20.18,p\;<\;0.001,\eta^{2}\;=\;0.7\right]$, with no interactions. Tones produced by finger-only actions (dotted line) were more deteriorated than tongue-only actions (black line). Combining both effectors (Figure 6B, dashed line) stabilized the CV for the slow tempo condition. A separate *post-hoc* pairwise *t*-test for the slow tempo condition confirmed that finger-only actions were significantly different from combined tongue-finger actions (Bonferroni: *p* < 0.01) in the slow tempo. However, in the medium and the fast tempo conditions, there was no longer a significant difference in the variability of combined tongue-finger actions and finger-only actions (Bonferroni: *p* = 1.0). With faster tempi, finger actions overrule the timing of the tongue.

In comparison to the previous study on the saxophone (Hofmann and Goebl, 2014), the tongue-only timing of the clarinettists showed a much higher temporal precision (clarinetist's mean CV = 0.074; saxophonists' mean CV = 0.11). We assume that the smaller mouthpiece of the clarinet, with a small tip opening (0.75 mm for Clarinet Maxton NA-1) might help to facilitate a more precise tonguing technique. However, the overall observed trends were similar in both studies. Combined tongue-finger actions improved the timing in the slow tempo condition. Conversely, in the medium tempo and in the fast tempo, the timing of the fingers again overruled the timing of the tongue. This indicates that the fingering technique plays a dominant role in the timing precision on woodwind instruments, even for playing techniques where the tongue initiates the tone.

### 3.4. Finger forces in the technical exercise task

**Figure 7:**
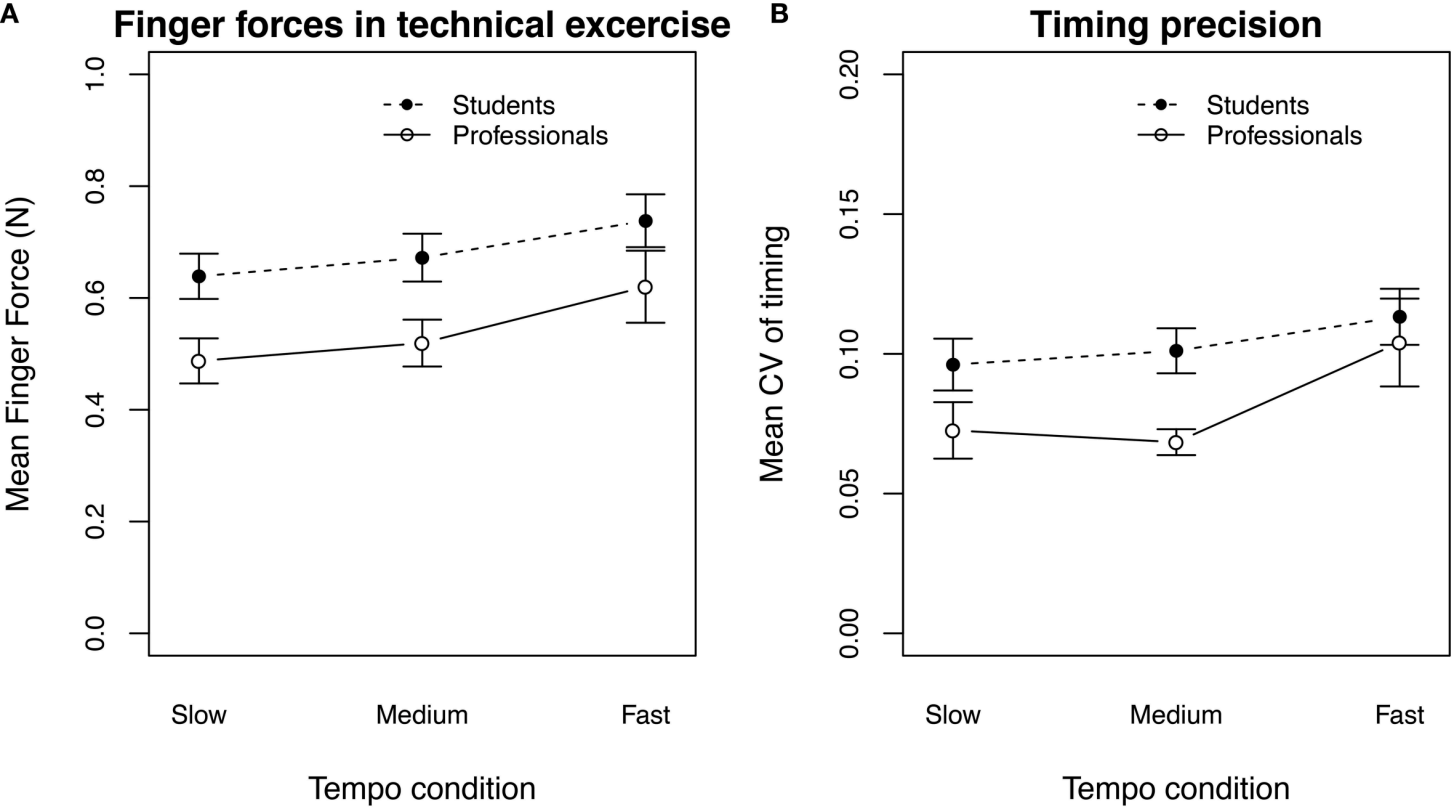
**Finger forces (A) and coefficient of variation (B) in the technical exercise, averaged by the tempo condition and the professional level of the participants**. The error bars show the standard error of the mean.

Looking at the measured left-hand finger forces for the legato sequence of the technical exercise task, participants applied very light finger forces to the sensors (*F*_*mean*_ = 0.64 N) in comparison to the performance task. Comparing the finger forces of the professional players and the clarinet students showed no significant difference [ANOVA *F*_(1, 20)_ = 1.653, *p* = 0.213], but a small effect (η^2^ = 0.276, Figure 7A). Finger forces of professional players were smaller (*F*_*mean*_ = 0.54 N) than to those of the students (*F*_*mean*_ = 0.68 N), especially in the slow and the medium tempo. Separate *post-hoc t*-tests (Bonferroni) confirmed this observation for the slow tempo condition (*p* < 0.05) and the medium tempo condition (*p* < 0.05), but not for the fast tempo condition (*p* = 0.153). A two-way repeated measures ANOVA on the timing precision by tempo as within-subjects factor and professional level as between-subjects factor showed no significant effect for the tempo condition [*F*_(2, 40)_ = 2.049, *p* = 0.142] or the professional level of the players [*F*_(1, 20)_ = 3.151, *p* = 0.0911, Figure 7B]. The same ANOVA on the timing error showed an effect of the tempo condition [*F*_(2, 40)_ = 16.751, *p* < 0.001], but also no significant effect of the professional level [*F*_(2, 20)_ = 1.653, *p* = 0.213].

### 3.5. Touch quality in the technical exercise task

In piano playing the way how players touch the keys (e.g., *pressed touch* vs. *struck touch*) influences the sound and the timing of the performance (Goebl and Palmer, 2008; Goebl et al., 2014). Also in clarinet performance, different types of touch were observed through motion capture measurements. The proportion of key depression and key releases that contained identifiable peak acceleration with the index finger were found to correlate with the temporal accuracy of the performances (Palmer et al., 2009b). In our clarinet finger force curves we looked for different touch types. In the force curves shown in Figure 2 one can see in the fourth panel (left-hand ring finger) that a spike occurs when the finger closes the tone hole. Similar to the acceleration peak for a *struck touch* in piano performance, this force peak indicates a certain clarinet touch quality where the finger strikes the sensor. In contrast, the second panel (left-hand index finger from the same player) does not show these spikes. In this case, the finger is pressing the sensor with a *pressed touch*. We detected the spikes in the finger force signal around each note onset landmark (−40 ms, + 100 ms), if they were larger than 85% of their neighboring values in a moving window with the size of 200 samples.

**Figure 8:**
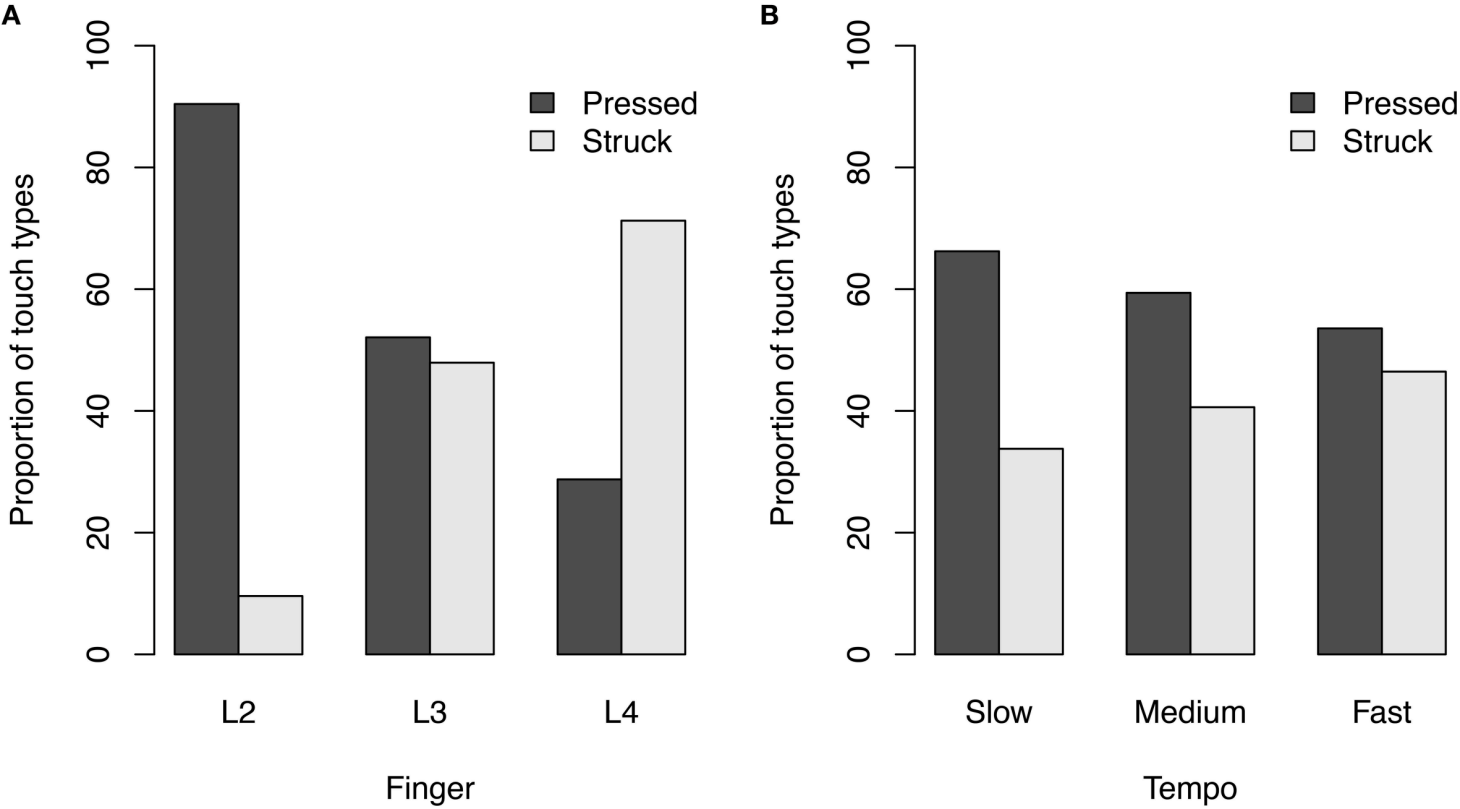
**(A)** The proportion of touch types in the technical exercise task for the individual fingers (L2 = index finger, L3 = middle finger, L4 = ring finger) of the left hand varied. **(B)** The proportions of touch types for three tempo conditions shows that with faster tempi the number of *struck touches* increased.

In contrast to piano performance, where a certain touch quality was observed with all fingers of a player, we observed that there were the most *pressed touches* with the index finger and the most *struck touches* with the ring finger (Figure 8A). In clarinet playing, if a finger is not used to close a tone hole, the finger is usually held above the tone hole far enough away to avoid altering the pitch or the quality of the sound by accidentally closing part of the tone hole. In some special playing situations the finger might be required to slide between a tone hole and an adjacent key, without losing the contact to the instrument. For example, the left-hand index finger (L2) has to operate between the first tone hole and the A4 key located next to the tone hole. Although such a key combination did not occur in this melody, the finger trajectory of the index finger might be different from those of the other fingers resulting in a higher number of *pressed touches*.

**Figure 9:**
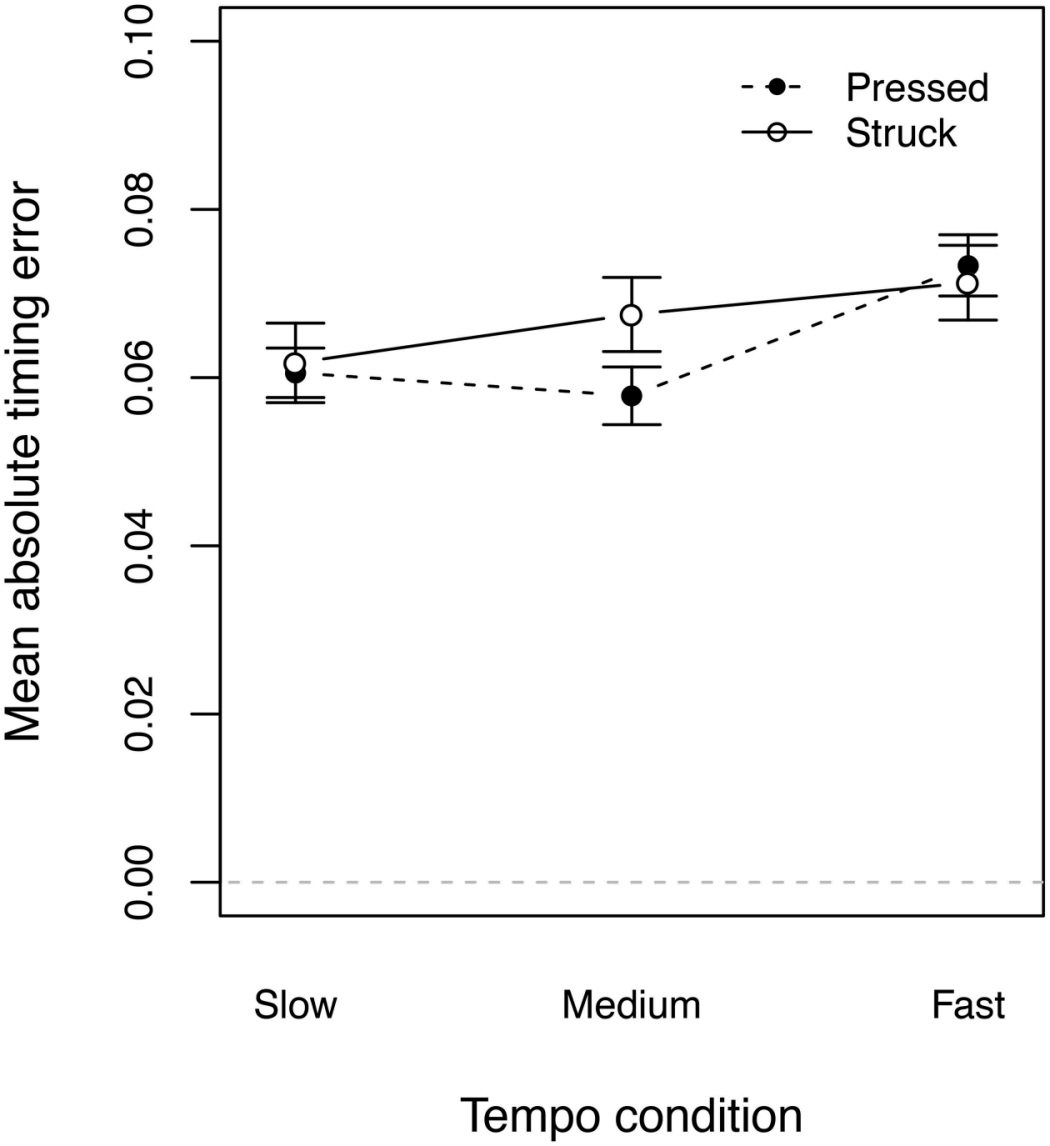
**Absolute timing error of successive note onsets, grouped by the touch type of the preceding note and the three tempo conditions of the technical exercise task**.

In the case of the Viennese clarinet, there are also ring-keys around the tone holes which are coupled to the key-work of the instrument. Springs are attached to the ring-keys to hold them away from the instrument body. In our technical exercise task, the index finger, the middle finger and the ring finger were closing the tone holes in a sequential fashion. Here, the impact of index finger hits the ring-key around the tone hole at first. The spring of the ring key might already damp the finger's impact, when it arrives at the sensor because the spring force works in the opposite direction. The same is true for the middle finger, but not for the ring finger. Here, the ring-key is coupled to the other ring-keys and already depressed when the finger closes the tone hole. Consequently only the ring finger is directly touching the sensor, where the other two fingers are decelerated by the ring-keys. Similar observation were made by Palmer et al. (2009b). Although their investigations were on the Boehm clarinet system (different key-work), they observed a different acceleration trajectory for the index finger than for the middle finger and the ring finger (right-hand) and explained it by only the index finger ring-key would move upon contact. Nevertheless, in our experiment the conditions of the clarinet were the same for all participants but we observed different touch types for the individual players, as well as for the three tempo conditions. The proportion of *struck touches* was significantly influenced by the tempo (χ^2^ = 216.26, *df* = 2, *p* < 0.001), showing more *struck touches* with increasing tempi for all players (see Figure 8B). However, we did not find a correlation between the proportion of *struck touches* and the mean absolute timing error of the following tone (*r* = 0.042, *p* = 0.64, see Figure 9)^2^.

**Figure 10:**
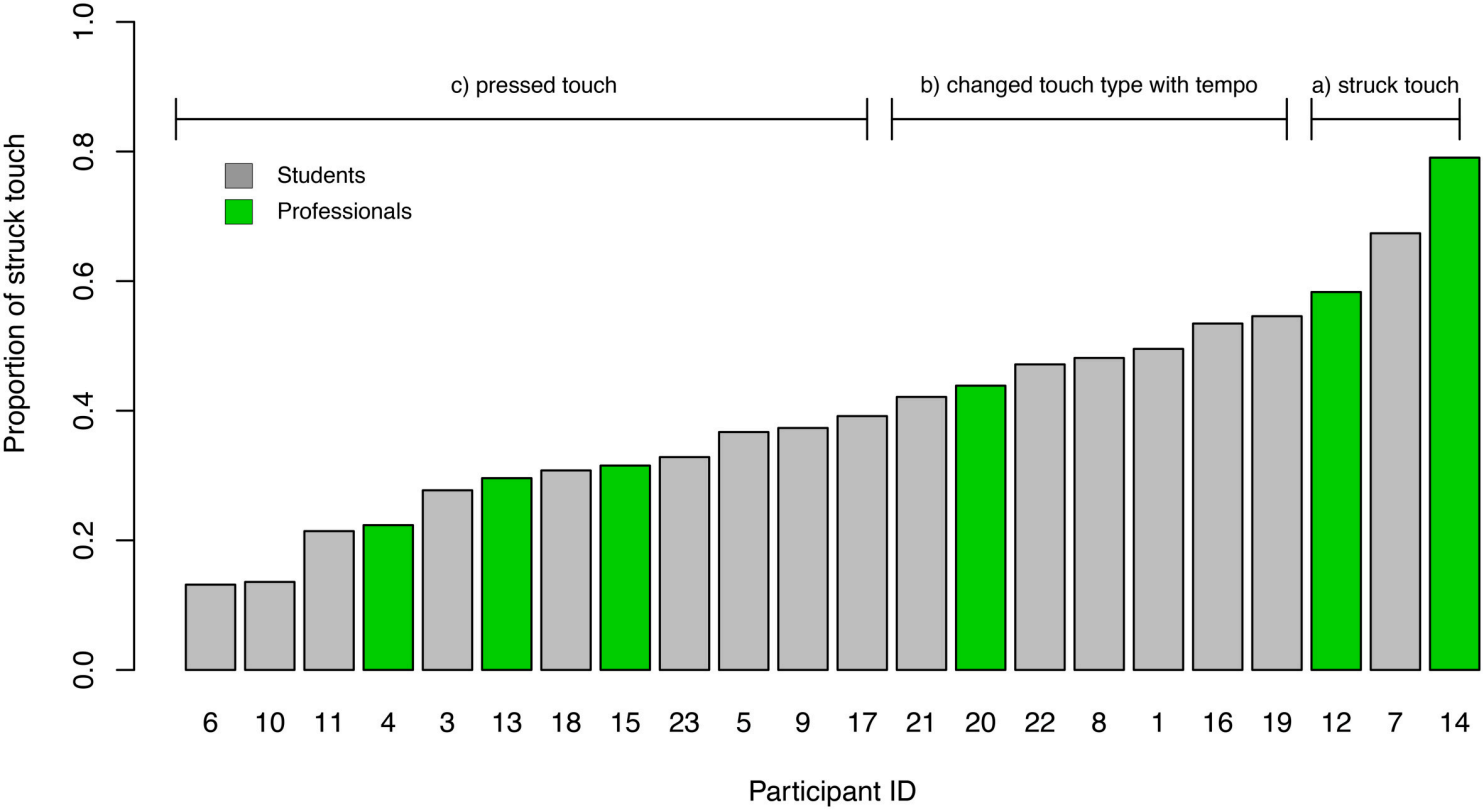
**Proportion of *struck touches* for each participant**. The gray bars indicate the participants who were students, the green bars label the professional performers. The participants were grouped by their *touch types*, according to the proportion of *pressed touch* and *struck touch* into three touch type groups.

**Figure 11:**
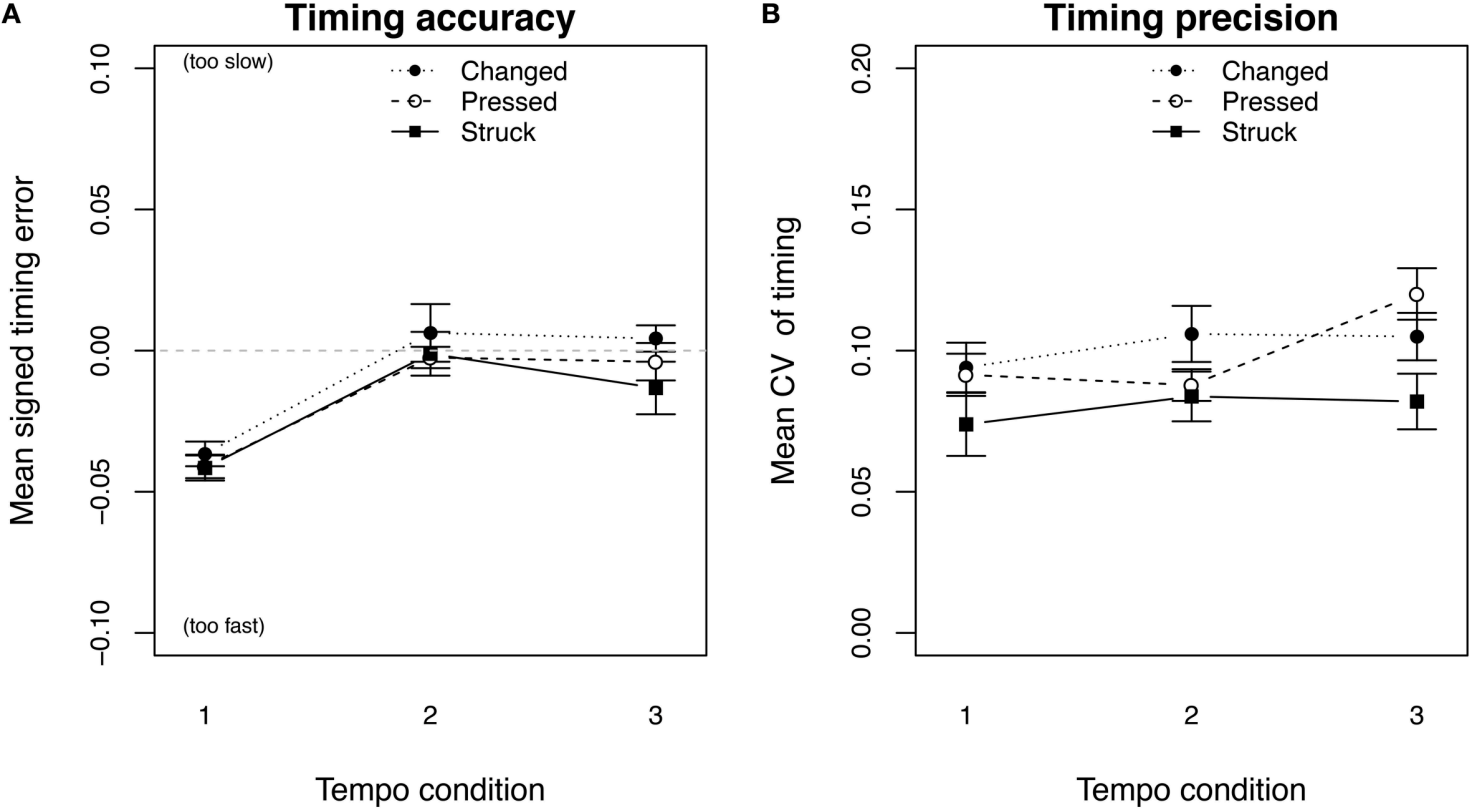
**Timing error (A) and coefficient of variation (B), for the finger-only condition (legato) of the technical-exercise task showed no significant differences in the timing for the three groups of players applying different *touch types***.

To further investigate a possible relationship between the touch type and the timing, we grouped our participants into three groups according to the proportion of the observed touch types: (a) players with more than 50% of *pressed touches* in every tempo condition, (b) players who changed their touch type to more than 50% of struck touches in the fast tempo, and (c) players with more than 50% of struck touches in all three tempo conditions. Figure 10 shows the three groups and the absolute proportion of the performed *struck touches*. The green bars show that the professional players did not entirely fall into one of the three groups. A two-way repeated measures ANOVA on timing error^3^ by tempo condition as within subjects and touch type group as between subjects showed a significant effect of the performed tempo condition [*F*_(2, 38)_ = 14.572, *p* < 0.001] but no significant effect of the touch type group [*F*_(2, 19)_ = 0.195, *p* = 0.825, Figure 11A]. The same ANOVA on the timing precision showed neither an effect for the tempo [*F*_(2, 38)_ = 2.009, *p* = 0.148] nor for the touch type group [*F*_(2, 19)_ = 0.74, *p* = 0.491, Figure 11B].

## 4. Discussion

Playing a musical instrument is a highly sophisticated motor task. Examining the complex motion patterns that professional performers use to master an instrument helps to understand how humans learn and coordinate such complex body movements.

With the sensor equipped clarinet we measured that clarinettists applied more finger force when they were playing expressively (*F*_*mean*_ = 1.21 N) than with a technical exercise task (*F*_*mean*_ = 0.64 N). This shows that even though clarinettists could only apply the minimal finger forces required to close the tone holes for pitch change, there are effects of the dynamics (more force in loud passages), the tempo (more force with slower tempi) and the register (more force in the high registers) when playing actual music. This suggests that primarily in a practice situation, the performers focus to such an extent on their playing technique that they apply only minimal finger forces to the tone holes, as recommended by clarinet teachers like Wehle (2007).

However, when performing expressively, multiple aspects influence the body motions of the performers and seem to affect the finger forces as well. Wanderley et al. (2005) observed that clarinettists make use of various expressive body movements during their performances. Clarinettists are stepping with their feet, bending their knees and waist, and also move their arms, move their head and their shoulders which results in movements of the clarinet. These so called *ancillary gestures* are not essential to produce the sound, but are found to be an important part of the performance quality, because they are linked to the mental representation of the musical piece. Wanderley demonstrated that with repetitions of the same piece, each performer showed similar movement patterns in relation to the musical structure. Immobilizing the performers immediately altered the timing of the performances. Furthermore, the visual aspect of a music performance also contains valuable information for the observing audience about the structure of the music, the emotional content as well as the professional level of the performers (Tsay, 2013).

As these *ancillary gestures* directly interact with the clarinet, we suggest that this also affects the finger forces and explains the effect of varying finger force profiles in expressive music performance. Furthermore, some of the *ancillary gestures* may be responsible for the technical problems in the finger force measurements. Although the sensors were especially designed for force measurements at the tone holes (Weilguni, 2013), unexpected forces occurred that caused damage on some sensor cells during this study. These damages happened when the performers were sliding between the keys or applied a torsional motion to the force sensors through flapping their arms.

With the technical exercise task we repeated an experiment we did earlier on the saxophone, where we looked into the timing produced with different combinations of tongue actions and finger actions (Hofmann and Goebl, 2014). We found similar trends in both studies, indicating that the fingers play a dominant role in the overall timing of single-reed woodwind performance. This is in line with the recommendation of clarinet teachers to study the finger movements first, even for exercises which actually focus on tonguing techniques (Mauz, 2011). From the force measurements we were not able find a correlation between the finger force profiles and the timing quality in the technical exercise task. The only indicator we found, was that the softest finger forces occurred with the professional players (*F*_*mean*_ = 0.54 N). Taking the results from our questionnaire into consideration, we assume that these experienced players developed a highly efficient playing technique over years.

Musicians practice their instrument over a long time and this can lead to unilateral stress for the body which can cause overuse syndromes. Those participants who reported discomfort in relation to their clarinet playing applied slightly larger finger forces than the other players, however this trend was not significant, probably due to the small number of participants. Furthermore, our participants were well aware of how much finger forces they were applying to the instrument, according to the answers in the questionnaire. Moreover, the overall mean finger forces for expressive clarinet playing (*F*_*mean*_ = 1.21 N) were lower than the finger forces reported for other musical instruments like the violin (2.7 N, Kinoshita and Obata, 2009), the guitar (30–50 N, Hori et al., 2013), or the piano (> 5 N, Parlitz et al., 1998).

It may seem contradictorily that Palmer et al. (2009b) observed improved temporal accuracy for clarinet performances with higher magnitudes of finger acceleration, but we measured very soft finger forces in the technical (timing) exercise task, especially for the professional players. We assume, that the professional clarinettists are able to produce fast but very soft and efficient finger movements. To verify this assumption simultaneous measurements of the finger force and the finger acceleration are required. Motion capture recordings of sensor clarinet performances would allow to investigate the finger trajectories, the position of the hands and the arms together with the resulting forces to the instrument. Furthermore, it would be interesting for future research to compare the techniques of beginner and intermediate (high school level) clarinettists with the technique of professional players. This would also give the chance to study finger actions for cases where a participant can barely perform the tasks and has to correct fingering mistakes.

In this study on the clarinet, we demonstrated how professional clarinettists and semi-professional clarinettists (music students) make use of tongue and finger movements to control the instrument. Such sensor-equipped wind instruments help to understand fine motor actions which are difficult to be visually observed. Tools like the sensor-clarinet might also support clarinet teachers in the future to give better advise in teaching situations and to demonstrate even small differences between their playing technique and that of their students.

## Author contributions

AH, WG: Design of the experiment, AH: Accomplished experiment, AH: Data analysis, AH, WG: Writing of report.

## Funding

This research was supported by the Austrian Science Fund (FWF) grants: P 23248 and P 24546.

## Disclosure

Parts of this work have been published in the first author's PhD thesis (Hofmann, 2015).

### Conflict of interest statement

The authors declare that the research was conducted in the absence of any commercial or financial relationships that could be construed as a potential conflict of interest.

## Footnotes

^1^Details about the detection function can be found in Hofmann and Goebl (2014). We used the same detection algorithm to have comparable measures for clarinet and saxophone performances.

^2^The data set was restricted to only legato and portato performances.

^3^To focus on only the fingering technique, the dataset was restricted to the finger-only conditions (legato articulation).

## Supplementary material

All musical scores used in the experiments can be found in the Supplemental Materials.
