# Supplementary material for: Finger Forces in Clarinet Playing
Source: Front Psychol. 2016 Aug 4;7:1140. doi: 10.3389/fpsyg.2016.01140 (PMC4973398; doi:10.3389/fpsyg.2016.01140)
Supplement: Supplementary file 1 [file DataSheet1.pdf]

Supplementary Material: Finger Forces in Clarinet Playing

Alex Hofmann and Werner Goebl
*Correspondence:
Author Name: Alex Hofmann
hofmann-alex@mdw.ac.at

All scores were produced by the authors using MuseScore software (Ver. 1.2., `https://musescore.org`).

1 SUPPLEMENTARY TABLES AND FIGURES

Supplementary Table 1. Selection of excerpts from the Clarinet Concerto No.1 in F minor (Op. 73) for clarinet in Bb, from Carl Maria von Weber according to the $2 \times 2 \times 2$ (tempo: slow–fast, dynamics: piano–forte, register: low–high;) experimental design. The letters refer to the scores giving in Supplementary Figures 1 (A–D) and 2 (E–H).

	Slow Tempo		Fast Tempo	
	Soft Dynamics	Loud Dynamics	Soft Dynamics	Loud Dynamics
Low Register	C	A	B	H
High Register	E	F	G	D

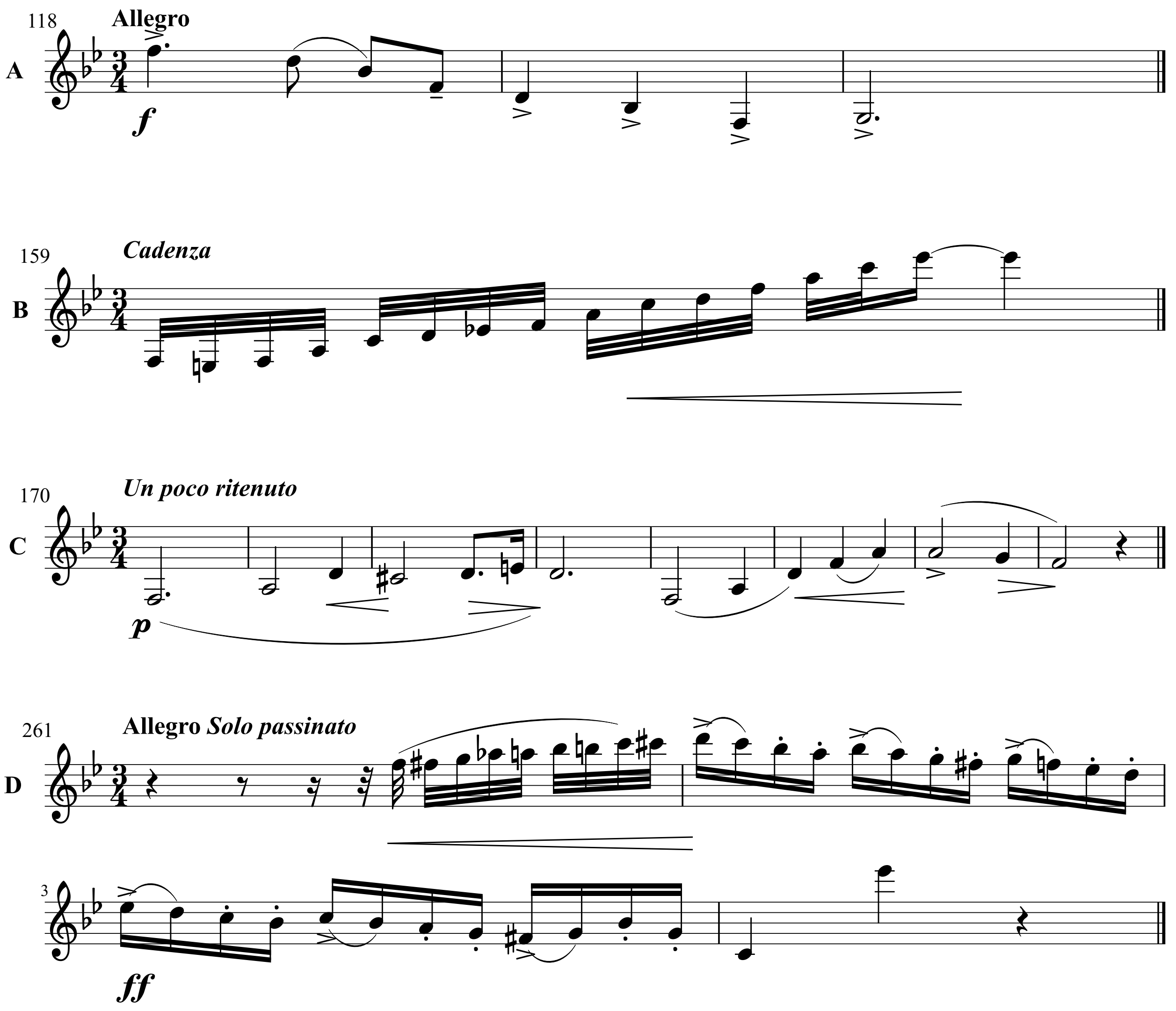

Supplementary Figure 1. Score excerpts from the Clarinet Concerto No.1 in F minor (Op. 73) for clarinet in Bb, composed by Carl Maria von Weber. The excerpts are giving in the order of appearance in the piece. Bar numbers refer to the position in the first movement.

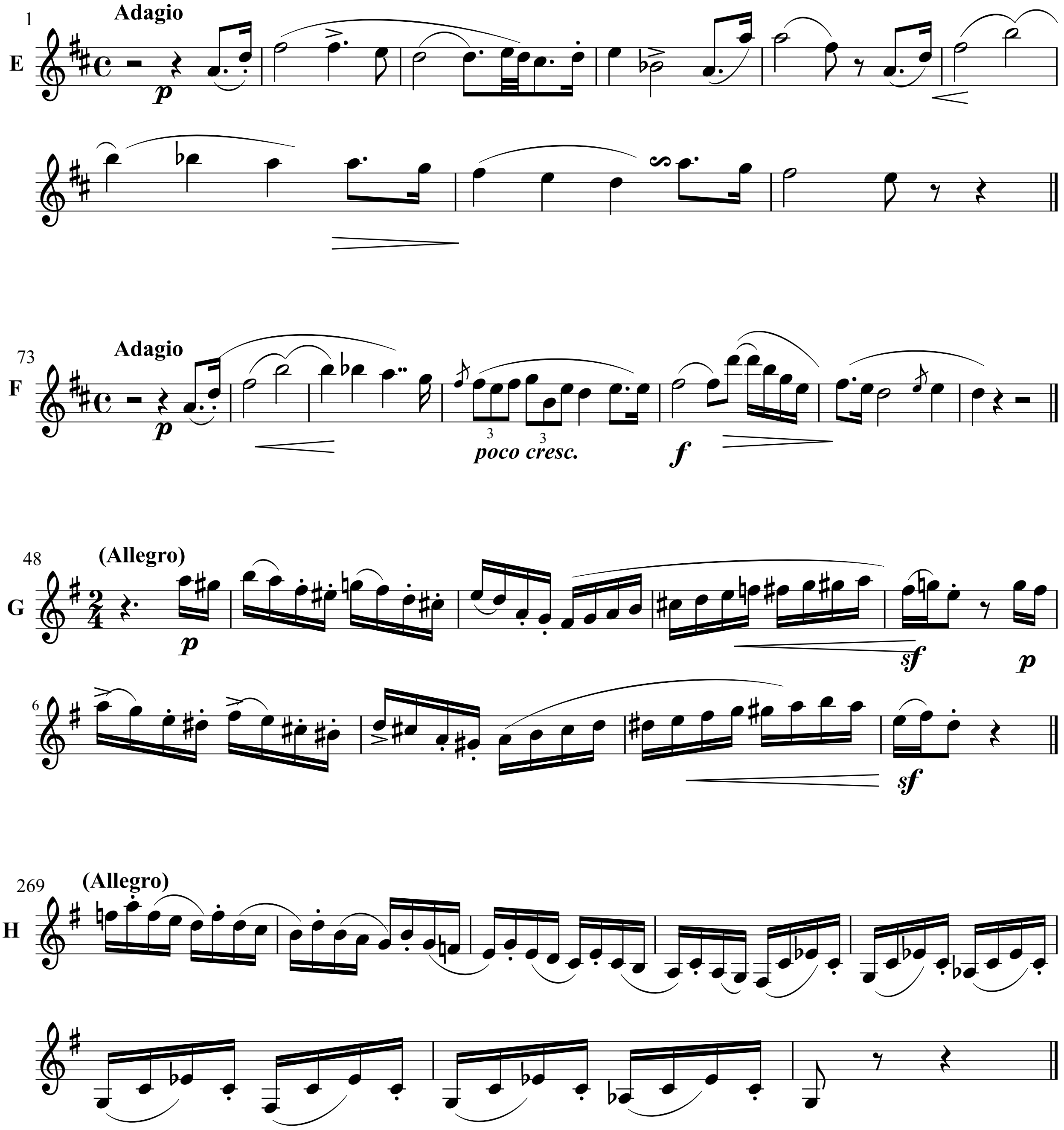

Supplementary Figure 2. Excerpts from the Clarinet Concerto No.1 in F minor (Op. 73) for clarinet in Bb, composed by Carl Maria von Weber. The bar numbers of the excerpts E) and F) refer to the position in the second movement, bar numbers of G) and H) refer the to third movement.

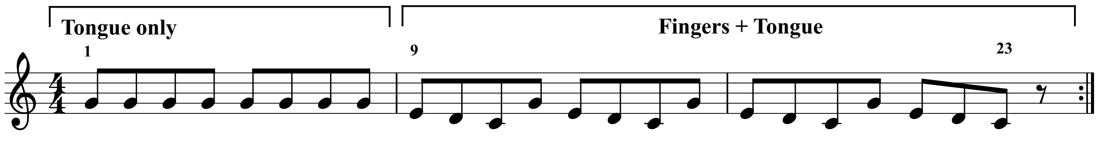

Supplementary Figure 3. The stimulus for the technical exercise task is a 23-tone melody in Bb-flat notation. Note numbers 1–8 are played with tongue actions only. Note numbers 9–23 require sequential left-hand finger actions to close the tone holes.
